# Severe Exertional Rhabdomyolysis in a Healthy 24-Year-Old Woman: A Case Report and Review of Literature

**DOI:** 10.7759/cureus.73545

**Published:** 2024-11-12

**Authors:** Jonathan Willard, Kelsey Green, Tenaadam Tsega, Srilekha Bathi, Miriam B Michael, Anand Deonarine

**Affiliations:** 1 Internal Medicine, Howard University Hospital, Washington, DC, USA; 2 Internal Medicine, University of Maryland, Baltimore, USA

**Keywords:** aki, exercise, exertional rhabdomyolysis, hyperkalemia, rhabdomyolysis

## Abstract

Rhabdomyolysis is characterized by the release of muscle cell components into circulation following muscle cell injury. Common causes include trauma and compression, exposure to drugs and toxins, and intense physical exercise. This study depicts a case of exercise-induced rhabdomyolysis following a cycling class. A 24-year-old African American woman presented to the emergency department with a one-day history of bilateral lower extremity myalgia, weakness, and stiffness, predominantly on the right side. She had participated in a one-hour morning cycling exercise class the previous day and came to the emergency department the following morning after noting dark-colored urine. Her initial creatine phosphokinase (CPK) level was 53,601 IU/L, leading to a diagnosis of exertional rhabdomyolysis. Her CPK continued to rise, peaking at 175,294 IU/L approximately 34 hours after admission. Serum chemistry, liver function, and clinical complications were closely monitored during the patient’s hospitalization. The patient responded well to IV fluids, showed clinical improvement, and did not require additional interventions or specialist consultations. Exertional rhabdomyolysis is increasingly common following exercise and may lead to serious complications. Prognosis in rhabdomyolysis is best when treated early and aggressively.

## Introduction

Rhabdomyolysis is characterized by the release of muscle cell components into circulation following muscle cell injury [1-3]. The term "rhabdomyolysis" originates from the Greek words rhabdos (rod-like/striated), mus (muscle), and lusis (breakdown) [1]. The diagnosis is confirmed when the serum creatine kinase level is >1000 U/L or at least five times the upper limit of normal. The pathogenesis of rhabdomyolysis appears to be due to a combination of mechanical and thermal muscle injury and adenosine triphosphate depletion. Causes of rhabdomyolysis can be classified into acquired and genetic categories. Acquired causes include trauma and exertion, hypoxic injury, infections, hyperthermia, and drug or toxin exposure. Genetic causes involve enzyme deficiencies affecting carbohydrate or lipid metabolism, as well as myopathies [1,2].

Exertional rhabdomyolysis, also commonly referred to as exercise-induced rhabdomyolysis, is increasingly common following exercise [2,4,5]. Strenuous muscular activity, such as sporadic strenuous exercise, as well as seizures and/or status epilepticus, can lead to rhabdomyolysis. Untrained individuals who engage in heavy weightlifting or exercise in extreme heat and humidity are at risk [2]. Factors predisposing individuals to exertional rhabdomyolysis include hypokalemia, extreme heat and humidity, sickle cell trait, exercise-induced asthma, and pre-exertion fatigue.

The common symptoms and signs of rhabdomyolysis are muscle weakness, pain/myalgia, and local swelling and may be associated with dark red color urine/myoglobinuria. The severity of the condition can range from mild elevations in creatine phosphokinase (CPK) to medical emergencies, such as compartment syndrome (CS), intravascular fluid depletion, disseminated intravascular coagulation (DIC), pigment-induced acute kidney injury (AKI), and cardiac arrhythmias [2,3]. Here, we present a case of exercise-induced rhabdomyolysis following a cycling class.

## Case presentation

A 24-year-old African American woman presented to the emergency department with a one-day history of bilateral lower extremity pain, swelling, and weakness, predominantly on the right side. She reported participating in a one-hour spinning exercise class for the first time that morning, followed by prolonged walking for the remainder of the day. She reported limited hydration the day before presenting to the emergency department. She began experiencing fatigue and a dull, aching pain in her bilateral lower extremities. The pain gradually worsened overnight, which she described as a constant, non-radiating, squeezing pressure (5/10 in severity) in her bilateral thighs. She presented to the emergency department the following morning after noticing dark-colored urine, which she described subjectively as “tea-colored.” She denied any alleviating factors and that the pain worsened with palpation of the lower extremity and ambulation. She has had no recent trauma and denied fever, chest, pain, or shortness of breath. She has no significant past medical history, no surgical history, and a family history significant for rheumatoid arthritis in her paternal grandmother. The patient denied alcohol consumption and the use of herbal supplements, cigarettes, tobacco products, and illicit drugs. Vital signs were within normal limits. Pertinent findings on physical examination included swelling of the bilateral thighs (right > left), with firmness on palpation and moderate tenderness.

**Figure 1:**
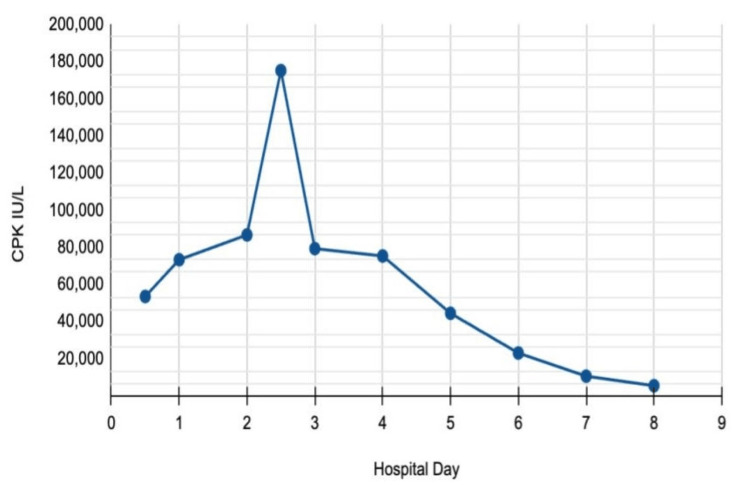
CPK trend CPK: creatine phosphokinase.

**Figure 2:**
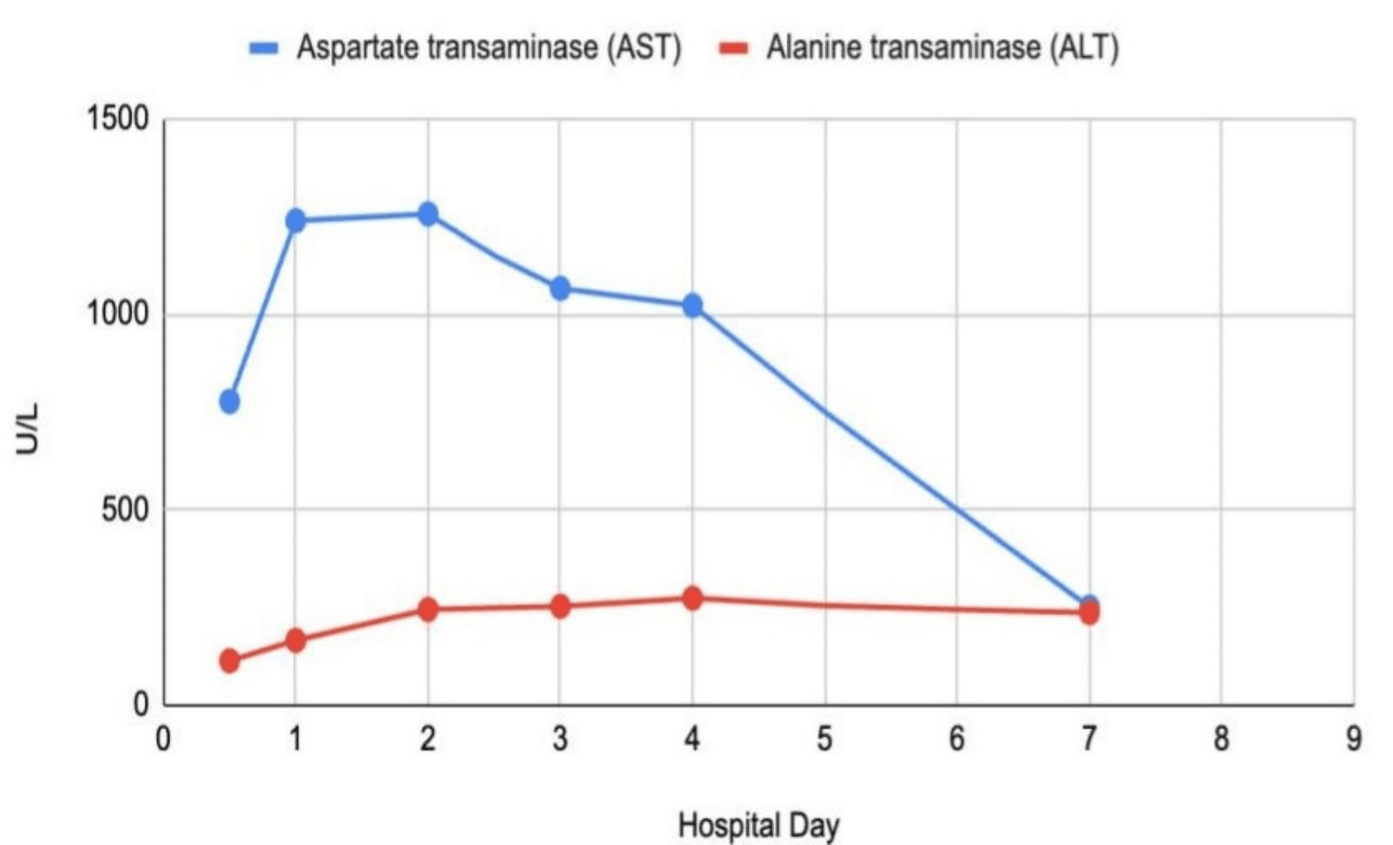
Aspartate transaminase and alanine transaminase trends

Lab orders included CPK, complete blood count, and comprehensive metabolic panel. Her initial CPK level was 53,601 IU/L, leading to a diagnosis of exertional rhabdomyolysis (Figure 1). The patient was started on IV fluids with normal saline at 250 mL/hour and was admitted to the medical floor. She also received acetaminophen for pain management. Initial pertinent lab results included an alanine transaminase (ALT) level of 114 U/L, an aspartate transaminase (AST) level of 777 U/L, and a lactate dehydrogenase level of 857 U/L (Figure 2). The initial urinalysis showed a dark brown urine, positive for ketones. A hepatitis panel and an abdominal ultrasound were ordered to assess liver function during her admission. The hepatitis panel returned negative results, and the abdominal ultrasound indicated a contracted gallbladder with no gallstones, a normal common bile duct, and a normal liver.

**Figure 3:**
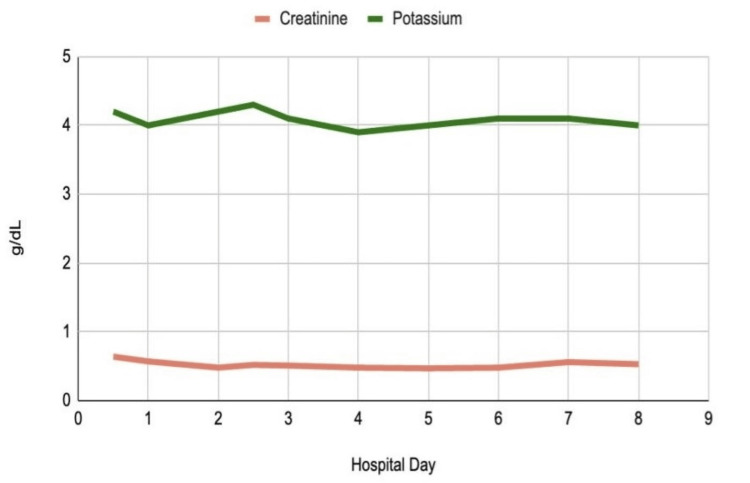
Creatinine and potassium trends

During her seven-day hospital stay, the patient’s CPK continued to increase for the first 36 hours. Approximately 34 hours after admission, her CPK peaked at 175,294 IU/L. It then began to downtrend on day 2 and was monitored daily thereafter. The patient also exhibited a mildly decreased calcium level of 7.2 mg/dL on day 2 and an increased phosphorus level of 4.8 mg/dL on day 5. ALT and AST levels gradually increased, reaching peak values of 274 U/L and 1256 U/L, respectively (Table 1). Kidney function was closely monitored throughout the patient's stay with serum creatinine and blood urea nitrogen (BUN) measurements (Figure 3). Daily monitoring for symptoms of CS and AKI revealed no acute signs based on both laboratory results and clinical presentation.

**Table 1:** Laboratory values over time ALT: alanine transaminase, AST: aspartate transaminase, BUN: blood urea nitrogen.

Variable	Normal range	2/20 08:17	2/20 13:17	2/21 06:20	2/21 18:03	02/22	02/23	02/24	02/25	02/26	02/27
CPK	35-230 IU/L	53,601	73,389	86,771	175,294	79,371	75,413	44,560	23,139	10,760	5,539
ALT	0-55 U/L	114	166	245	N/A	253	274	N/A	N/A	237	N/A
AST	0-50 U/L	777	1239	1256	N/A	1066	1022	N/A	N/A	251	N/A
Creatine	0.6-1.1 mg/dL	0.64	0.57	0.48	0.52	0.51	0.48	0.47	0.48	0.56	0.53
BUN	7-25 mg/dL	9	7	5	5	4	5	5	6	7	6
Calcium	8.5-10.3 mg/dL	8.7	7.2	7.5	7.9	7.6	7.6	7.8	7.9	8.4	7.7
Potassium	3.5-5.0 mg/dL	4.2	4.0	4.2	4.3	4.1	3.9	4.0	4.1	4.1	4.0

The patient responded positively to IV fluids, showed clinical improvement, and did not require additional interventions or specialist consultations. She was discharged on hospital day 7. Two weeks later, she presented for a follow-up visit in the clinic with no clinical signs of rhabdomyolysis or any related complications. Her vital signs and laboratory tests were within normal limits, and she was advised to establish primary care and follow-up as needed.

## Discussion

This case report highlights a 24-year-old woman who developed exertional rhabdomyolysis due to a lack of conditioning in aerobic training. There has been a notable increase in the number of patients hospitalized for exertional rhabdomyolysis. While it was once primarily associated with endurance exercise like long-distance running, recent studies indicate a shift toward associations with eccentric training, such as CrossFit, and aerobic training [5,6]. Spinning classes have also been shown to increase the incidence of exercise-induced rhabdomyolysis, with significantly higher CPK levels on admission and peak measurements [7]. Furthermore, cases of rhabdomyolysis following low-intensity and low-load, high-repetition exercises have been documented, posing challenges in diagnosis and treatment [3,8].

Rhabdomyolysis is caused by the breakdown and necrosis of skeletal muscles, which releases muscle cell components into the bloodstream, potentially leading to AKI. The mainstay laboratory finding for diagnosing rhabdomyolysis is a CPK value of 1000 or greater. CPK values typically rise within the first 12 hours, peak within 24-72 hours, and normalize within five days of injury [9]. Urine myoglobin, electrolytes, clinical presentation, and history are also useful diagnostic tools [10]. Intravenous fluid resuscitation with normal saline is the standard treatment; however, patients with rhabdomyolysis may also benefit from fluids combined with sodium bicarbonate, sodium chloride, or potassium chloride [11]. Prognosis is best when treatment is initiated early and aggressively with fluid administration and appropriate electrolyte correction [9].

Several other etiologies may lead to elevated CPK and a diagnosis of rhabdomyolysis. Crash injuries, resulting from physical trauma, require immediate attention and treatment, as they can lead to life-threatening complications such as hypotension, rhabdomyolysis, and AKI [12]. Acute alcohol intoxication may cause liver dysfunction and skeletal muscle injury, thus inducing nontraumatic rhabdomyolysis and AKI [13]. Other common causes of nontraumatic rhabdomyolysis, especially in hospitalized patients, include the use of prescription and over-the-counter medications as well as the use of illicit drugs [14]. Seizures may also precipitate rhabdomyolysis, with literature supporting hyponatremia following seizures as a key pathogenic factor [15]. Additionally, various viruses, including COVID-19, influenza, and adenovirus, have been implicated as potential triggers of rhabdomyolysis [16].

Several life-threatening complications may be direct consequences of rhabdomyolysis. AKI may be a common complication leading to a clinical presentation, including pigmented granular casts, brown or red urine, and rapidly increasing creatinine [17]. AKI may result in dangerous electrolyte derangements, specifically hyperkalemia. Hyperkalemia in the setting of rhabdomyolysis may cause early echocardiogram changes such as shortened QT intervals, peaked T waves, and ST depressions, which lead to potentially fatal cardiac dysrhythmias [18]. Other severe complications of rhabdomyolysis include CS, in which increased intramuscular pressure causes local ischemia, and DIC, where the destruction of muscle cells activates the coagulation cascade, stimulating intravascular coagulation [19,20].

## Conclusions

This study presents a case of bilateral lower extremity rhabdomyolysis following a one-hour cycling class the morning before presentation. This case emphasizes the link between exertional rhabdomyolysis and aerobic exercises, such as spinning classes. The patient's significantly elevated CPK levels upon admission highlight the importance of early medical attention to prevent complications like AKI. In addition to early medical care, proper conditioning and hydration are crucial preventive measures. In conclusion, as eccentric and aerobic training gain popularity, documenting cases of exertional rhabdomyolysis is essential to identify common risk factors and better understand this condition.
